# Blood Mercury Levels of Zebra Finches Are Heritable: Implications for the Evolution of Mercury Resistance

**DOI:** 10.1371/journal.pone.0162440

**Published:** 2016-09-26

**Authors:** Kenton A. Buck, Claire W. Varian-Ramos, Daniel A. Cristol, John P. Swaddle

**Affiliations:** 1 Institute for Integrative Bird Behavior Studies, College of William and Mary, Williamsburg, Virginia, United States of America; 2 Biology Department, Colorado State University – Pueblo, Pueblo, Colorado, United States of America; Fred Hutchinson Cancer Research Center, UNITED STATES

## Abstract

Mercury is a ubiquitous metal contaminant that negatively impacts reproduction of wildlife and has many other sub-lethal effects. Songbirds are sensitive bioindicators of mercury toxicity and may suffer population declines as a result of mercury pollution. Current predictions of mercury accumulation and biomagnification often overlook possible genetic variation in mercury uptake and elimination within species and the potential for evolution in affected populations. We conducted a study of dietary mercury exposure in a model songbird species, maintaining a breeding population of zebra finches (*Taeniopygia guttata*) on standardized diets ranging from 0.0–2.4 μg/g methylmercury. We applied a quantitative genetics approach to examine patterns of variation and heritability of mercury accumulation within dietary treatments using a method of mixed effects modeling known as the 'animal model'. Significant variation in blood mercury accumulation existed within each treatment for birds exposed at the same dietary level; moreover, this variation was highly repeatable for individuals. We observed substantial genetic variation in blood mercury accumulation for birds exposed at intermediate dietary concentrations. Taken together, this is evidence that genetic variation for factors affecting blood mercury accumulation could be acted on by selection. If similar heritability for mercury accumulation exists in wild populations, selection could result in genetic differentiation for populations in contaminated locations, with possible consequences for mercury biomagnification in food webs.

## Introduction

Mercury is a global pollutant that can diminish reproductive success and have many other negative effects on wildlife, across a range of exposures [1–3]. Anthropogenic emissions of mercury have increased by approximately three-fold over the last 200 years [4]; though declines have been reported recently in some areas [5]. In its methylated form mercury readily enters food webs and can biomagnify to toxic levels at the top of the food web, such as in many bird species [2,6,7]. While the lethal and sublethal effects of mercury toxicity have been well-studied in fish-eating species, terrestrial songbirds are also at risk from mercury contamination [8].

Mercury reduces reproductive success through a number of mechanisms including embryonic toxicity [9,10], endocrine disruption [11], and/or changes in behavior of parents [1,12,13], and has been associated with reduced reproductive success in a variety of wild bird species [14–17]. A series of experiments on Mallards (*Anas platyrhynchos*) and Black Ducks (*Anas rubripes*) have shown decreased production of young after mercury exposure [10,18–21]. The only large-scale captive dosing study of songbirds to date demonstrated a reduction in fledgling production between 16% and 50% for zebra finches (*Taeniopygia guttata*) exposed to dietary mercury concentrations ranging between 0.3 and 2.4 μg/g (parts per million, hereafter, ppm) mercury [3,22].

Because mercury exposure affects reproduction and many factors contributing to survival in birds [3,8,23], selection may favor individuals who are more tolerant to mercury. In order for any adaptation to occur, however, there must be a genetic component to phenotypic variation for selection to act upon. Therefore, in order to estimate the potential for evolution to occur within a population, it is necessary to not only measure the strength of selection (e.g. the relationship between mercury levels and differences in reproductive success), but also the genetic variances affecting the focal phenotypic trait (e.g. mercury levels) [24]. The amount of genetic variation and the degree to which it contributes to phenotypic variation (heritability) is measured by comparing phenotypic resemblance among individuals of known relatedness [25].

If variation in response to mercury exists at the population level, and if it can be attributed to heritable genetic differences, a population-level response to mercury may evolve. Mercury tolerance in bacteria is widespread among both Gram-positive and Gram-negative bacteria [26]. The majority of studies examining evolutionary and genetic response to mercury in animals have measured tolerance in aquatic invertebrates [24,27–35] and a smaller number of terrestrial invertebrates [36]. Mercury tolerance has been evaluated in few vertebrates. To our knowledge it has only been described in fish [37,38] and in a previous study where we demonstrated reproductive differences among families of zebra finches exposed to mercury [22]. We are not aware of any published studies of the heritability of traits related to mercury accumulation or tolerance in a terrestrial vertebrate species. Given the increased awareness of mercury-exposure in terrestrial vertebrates, it is important to assess their capacity to evolve resistance to mercury contamination. Additionally, most studies related to mercury tolerance have investigated the mechanisms underlying tolerance directly, with relatively little consideration of genetic variation in tolerance [26,31], or have measured the response to selection directly in laboratory or wild settings [39]. Terrestrial birds are increasingly exposed to environmental mercury and exposure is likely to increase due to global climate change [40]. The cellular and physiological mechanisms that determine variance in mercury tolerance could be very different in birds compared with fish, invertebrates, or bacteria. Thus, there is need to understand genetic variance of mercury tolerance-related traits in bird species. Such information will help us to evaluate better the potential for adaptation to environmental mercury in birds.

We performed a captive-dosing study using a model songbird, the zebra finch, maintained on a diet containing environmentally-relevant concentrations of mercury. Because both likely pathways of mercury detoxification in birds, including excretion into feathers and eggs [41–45] and demethylation in the liver and kidneys [6,46,47], reduce levels of mercury circulating in the blood, we used mercury accumulation in blood as a representative phenotype for traits associated with mercury excretion and detoxification. We evaluated the potential for adaptive response to mercury in our population of captive-dosed zebra finches by measuring phenotypic variation and heritability of blood mercury accumulation. Similar quantitative genetic approaches have been applied to study the evolution of resistance to other ecotoxins [24], but there has been limited application to the evolution of mercury tolerance in vertebrates, and none in birds. There have also been studies of quantitative genetics of morphology and coloration in zebra finches [48,49], but at much smaller sizes than included in our large breeding design.

## Materials and Methods

### Study population and dietary mercury treatment groups

Zebra finches are commonly used in laboratory and field studies [50,51]. Their genome has been fully sequenced [52] and the ease with which they can be bred and maintained in captivity has made them a model for studies of avian physiology, behavior, development, and evolution [53,50]. Zebra finches have recently emerged as a system for captive toxicological studies [3,22,54–61].

All research was conducted from August 2010—May 2012, and this study was approved by The College of William and Mary's Institutional Animal Care and Use Committee (IACUC 2012-05-23-7982). A parental generation of 180 individuals (90 males, 90 females) was randomly selected from a genetically diverse captive population of zebra finches whose pedigree was recorded for at least one generation before the onset of this experiment. Five treatment groups (18 pairs per treatment) were maintained on a pelletized finch food (Zupreem FruitBlend, Shawnee, KS) and randomly assigned to one of five dietary mercury concentrations (0.0, 0.3, 0.6, 1.2, 2.4 ppm wet weight, equivalent to dry weight concentrations of 0.0, 0.35, 0.70, 1.39, 2.79 ppm methylmercury cysteine). We did not use any control group birds in subsequent analyses as they had almost undetectable levels of mercury in their blood, as in our other studies [3,22]. The lower doses of dietary mercury (0.3, 0.6 ppm) were selected to represent levels detected in insect food items found in mercury-contaminated habitats, such as the South River watershed in Virginia, USA [7]. Higher levels of exposure (1.2, 2.4 ppm) represented worst-case scenarios at highly contaminated sites [3]. Of note, in our review of prior quantitative genetics studies of zebra finches the largest sample size we could find was a study based on 32 breeding pairs [48]. As our study was based on 72 breeding pairs and included 2,641 individuals we feel that our same is large as well as being genetically diverse. All birds were maintained in standardized cages with *ad libitum* access to appropriate mercury-dosed or control food, vitamin-enriched water (Vitasol, Islandia, NY), oyster shell grit, cuttlefish bone, and perches, and maintained on a long day (14:10 L:D) photoperiod to encourage breeding.

Each treatment group was originally dosed in single-sex cages for a period of ten weeks until blood mercury levels had plateaued. Individuals were then paired at random within treatment, avoiding any inbreeding between known relatives, and pairs were bred continuously for one year. Breeding pairs were provided with a nest box and had access to nesting material. Because common environmental and maternal effects inflate estimates of genetic influence within broods [62], age- and size-matched broods were cross-fostered as nestlings when available (nestlings cross fostered, with mercury treatment as the subscript: *N*_0.3_ = 15; *N*_0.6_ = 15; *N*_1.2_ = 7; *N*_2.4_ = 0). After reaching fledged independence (approximately 50 days) offspring were transferred to one of five large, aviaries where they lived in groups with other young birds on appropriate mercury or control diets identical to what their parents had been fed.

### Food preparation

Mercury-dosed foods were prepared by homogenizing stock concentrations of methylmercury cysteine into pelletized finch food [3]. Selenium concentrations in the prepared finch food were negligible. Each batch of each diet was sampled 10 times for mercury content to ensure that it was within 10% of the nominal dose, while average mercury content in the control diet was 0.004 ± 0.002 ppm. Mercury-dosed diets contained between 99.27–102.13% of desired values with a mean concentration of 100.79% of the calculated wet weight mercury concentration.

### Quantification of mercury accumulation

Blood mercury content is a common measurement to assess overall mercury accumulation [23]. Blood mercury levels were tested weekly until asymptote and then monthly for each adult bird. Blood samples of approximately 20–50 μL were collected in 70μL heparinized capillary tubes after we used a 30-gauge needle to puncture the cutaneous ulnar vein to produce a surface droplet of blood. Each capillary tube was sealed with Crito-Caps (BD, Franklin Lakes, NJ), stored in an individually labeled 10cc BD Vacutainer, and frozen at -20°C until analysis.

All samples were analyzed, without drying, for total mercury content on a DMA-80 (Direct Mercury Analyzer, Milestone Scientific, Livingstone, NJ) using previously described methods [3]. The DMA-80 was calibrated every two months, or as needed, throughout the study. Quality assurance measures were maintained using two certified reference materials: dogfish muscle tissue and dogfish liver (DORM-3 and DOLT-4, National Research Council of Canada, Ottawa, ON, Canada). Each batch of samples was preceded and followed by the following sequence of quality control samples: empty system blanks (x2), empty receptacle method blank, DORM-3, DOLT-4, distilled water, system blanks (x3). Recoveries for certified reference materials were within accepted limits and averaged 103.48 ± 0.43% (n = 1489) for DORM-3 and 100.32 ± 0.22% (n = 1461) for DOLT-4. Matrix spikes with bird blood were performed regularly, and recoveries averaged 101.15 ± 3.56% (n = 62). The average calculated minimum detection limit was 0.008 ± 0.001 ppm. The relative percent difference for duplicate Hg samples from the period that the samples in this study were analyzed (n = 639 pairs of duplicates) was 2.61%.

In birds, total mercury concentration is a strong proxy for methylmercury concentrations. Specifically, more than 95% of the total mercury in avian eggs and blood consists of methylmercury [63,64], which has also been confirmed in our colony of zebra finches (D. A. Cristol, unpublished data). Further, individual variation in total mercury concentrations is very highly associated with individual variation in methylmercury concentrations, *r*^2^ = 0.99 [63]. Hence, we can infer patterns of variation in methylmercury, which is highly biologically active, by quantifying total mercury concentrations in individual birds.

We visually monitored all birds in this study daily for any adverse health effects, which we did not observe. Notably, the doses of mercury used here did not influence adult survival; all of the doses were intentionally sub-lethal. We do know that the immune system can be somewhat suppressed [54] and stress-induced corticosterone responses can be dampened [65] in the 1.2 and 2.4 ppm treatments, but we did not observe any obvious infections that affected the welfare of our birds.

### Quantitative genetics

We measured the phenotypic variation and heritability of blood mercury accumulation within dietary mercury treatments using a repeated-measures Animal Model. The Animal Model is a method of mixed modeling that partitions phenotypic variation for a quantitative trait into separate genetic and environmental variance components and includes an individual’s breeding value, or individual genetic merit, as a random effect [66]. Mercury levels in offspring included in the model were those obtained after birds reached maturity (approximately 100 days). All analyses were conducted using ASReml version 3 [67].

We ran Animal Models for each dietary mercury treatment separately with independent variance components partitioned for each. The initial models included sampling date, age, and sex as fixed effects, and random effects for each trait of additive genetic effect (variance *V*_*A*_), 'permanent environment' (V_PE_) [68,69], foster nest environment (V_F_), and residual effects (V_R_). As only some of the offspring were cross-fostered we minimized parent-offspring covariation due to early-life common environmental effects by including a permanent environmental effect in all models. Including such an effect lowers heritability estimates, which we confirmed by re-running all models without the permanent environmental effect. We feel it is justified to keep this permanent environmental effect in every model but recognize that this returns conservative estimates of heritability.

The model partitioned variance components for each random effect. Variation of blood mercury accumulation within treatments was measured by total phenotypic variance (*V*_*P*_), which was calculated as the sum of all variance components for each random effect plus the residual error (*V*_*P*_ *= V*_*A*_ *+ V*_*PE*_ *+ V*_*F*_*+ V*_*R*_). Between-individual variance (*V*_*IND*_) was calculated as the sum of additive genetic variance and permanent environmental variance (*V*_*IND*_ *= V*_*A*_ *+ V*_*PE*_). Repeatability (*r*^*2*^ *= V*_*Ind*_*/V*_*P*_), narrow-sense heritability (*h*^*2*^ *= V*_*A*_*/V*_*P*_), and permanent environmental effect (*pe*^*2*^ *= V*_*PE*_*/V*_*P*_) within each treatment were calculated as the proportion of the related variance component to total phenotypic variance. Comparisons between treatments were made using mean-scaled coefficients of variation for total phenotypic (*CV*_*P*_), permanent environmental (*CV*_*PE*_), foster environmental (*CV*_*F*_), and residual variances (*CV*_*R*_). All coefficients of variance were calculated as the square root of the respective variance component divided by the treatment mean of blood mercury. Two mean-scaled measures of evolvability (i.e., potential to evolve given the right circumstances or additive genetic variance), coefficient of additive genetic variation and its square (I_A_) [70,71] were calculated for all mercury dose treatments as $CV_{A}=\;\sqrt{V_{A}}/\bar{X}$ and $I_{A}=V_{A}/{\bar{X}}^{2}$, where $\bar{X}$ is the blood mercury treatment mean. Standard errors for coefficients of variation were calculated using previously described methods [70]. Statistical significance values for fixed effects were estimated using conditional Wald *F* statistics [67]; statistically non-significant effects (*p* > 0.05) were removed from the model, leaving only the main effect and other statistically significant interactions. The statistical significance of random effects was tested using the likelihood ratio test. The statistical significance of variance ratios (*r*^*2*^, *h*^*2*^, *pe*^*2*^) was calculated using one-tailed *t*-tests with the standard errors reported by ASReml [67].

## Results

**Fig 1:**
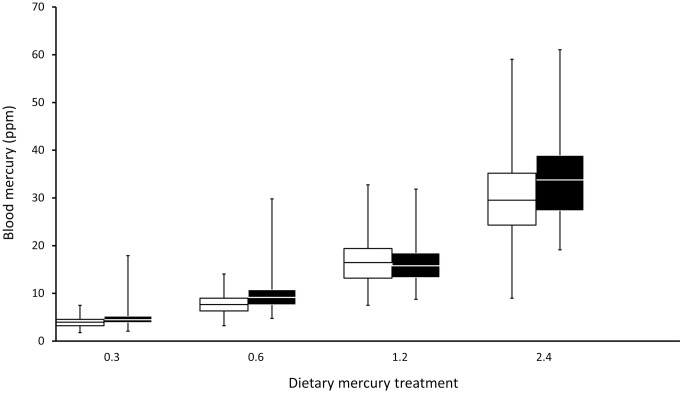
Blood mercury accumulation for each dietary dose of zebra finches. Parental generation values are depicted in clear bars to the left within each treatment group and offspring generation values are shown by the filled bars to the right of each pair in a treatment group.

Blood mercury accumulation exhibited considerable among-individual variation within all dietary mercury treatments (Fig 1). Mean-standardized estimates of variation, represented by coefficients of total phenotypic variation (*CV*_*P*_), were equivalent across all levels of dietary exposure and ranged from 0.239 to 0.283 (Table 1). Repeatability (*r*^*2*^) of individual blood mercury accumulation ranged from 0.200 to 0.458 and was highly statistically significant for all mercury treatments (Table 1).

**Table 1:** Variance estimates for each dietary mercury treatment.

Treatment	n	Mean	*CV*_*P*_ (SE)	*CV*_*A*_ (SE)	*CV*_*PE*_ (SE)	*CV*_*F*_ (SE)	*CV*_*R*_ (SE)	*pe*^*2*^ (SE)	*r*^*2*^ (SE)	*h*^*2*^ (SE)	*I*_*A*_
**0.3**	741	4.22	0.268	0.022	**0.118**	0	0.239	**0.193**	**0.200**	0.007	0.0005
		(1.20)	(0.076)	(0.085)	**(0.040)**		(0.068)	**(0.066)**	**(0.043)**	(0.053)	(0.032)
				*p* = 0.913	***p* = 0.002**			***p* = 0.002**	***p* < 0.001**	*p* = 0.449	
**0.6**	807	8.53	0.283	**0.192**	0	0	0.208	0	**0.458**	**0.458**	0.037
		(2.48)	(0.087)	**(0.069)**			(0.063)		**(0.116)**	**(0.116)**	(0.017)
				***p* < 0.001**					***p* < 0.001**	***p* < 0.001**	
**1.2**	582	16.5	0.239	0.139	0	**0.024**	0.192	0	**0.341**	**0.341**	0.019
		(4.34)	(0.066)	(0.052)		**0.119**	(0.053)		**(0.141)**	**(0.141)**	(0.010)
				*p* = 0.090		***p* < 0.001**			***p* < 0.001**	***p* < 0.001**	
**2.4**	511	30.81	0.259	0.042	**0.146**	0	0.210	**0.318**	**0.344**	0.026	0.002
		(7.99)	(0.068)	(0.084)	**(0.048)**		(0.055)	**(0.113)**	**(0.059)**	(0.102)	(0.007)
				*p* = 0.806	***p* = 0.002**			***p* = 0.003**	***p* < 0.001**	*p* = 0.401	

Sample size, mean, coefficients of variation, and variance ratios for blood mercury accumulation in zebra finches exposed to the four dietary mercury treatments. Refer to the text for explanation of coefficients of variation. Values are reported with standard error (SE); values in bold indicate p < 0.05.

The contribution of additive genetic variation on blood mercury accumulation was non-linear with increasing mercury exposure. Statistically significant (i.e. non-zero) heritabilities were calculated for the 0.6 and 1.2 mercury treatments (*h*^*2*^ = 0.458 and 0.341, respectively). Similarly, the mean-scaled measures of evolvability, coefficient of additive genetic variation (*CV*_*A*_) and *I*_*A*_, were highest and significantly non-zero for finches dosed at 0.6 ppm mercury but were lower for the 1.2 ppm mercury treatment (Table 1). High *CV*_*A*_ and *I*_*A*_ values indicate a notable degree of genetic influence on mercury accumulation and a greater evolutionary potential. Zebra finches dosed at 0.3 and 2.4 ppm mercury did not exhibit statistically significant contributions of additive genetic variance on blood mercury (Table 1), although the partitioning of among-individual variance into significant permanent environmental effects may have prevented an upward bias of additive genetic variation in these treatments (Table 1). When we removed the permanent environment effect from the models, all heritability estimates increased, significantly so in the 2.4 mercury treatment—to be comparable to the other treatments. However, the 0.3 mercury group still remained lower (Table 2). Common environmental effects, measured as foster environment (*CV*_*F*_), had a negligible effect in all treatments except in the 1.2 ppm dietary mercury dose (Table 1). *CV*_*F*_ was not estimated for the 2.4 treatment due to reduced nestling survival [3].

**Table 2:** Variance estimates when the permanent environment effect was included or not included in models.

	With permanent environment effect		Without permanent environment effect	
Treatment	*CV*_*A*_ (SE)	*h*^*2*^ (SE)	*CV*_*A*_ (SE)	*h*^*2*^ (SE)
0.3	0.022 (0.085)	0.007 (0.053)	0.125 (0.069)	0.210 (0.053)
0.6	0.192 (0.069)	0.458 (0.116)	0.220 (0.095)	0.529 (0.050)
1.2	0.139 (0.052)	0.341 (0.141)	0.139 (0.100)	0.341 (0.140)
2.4	0.042 (0.084)	0.026 (0.102)	0.161 (0.090)	0.354 (0.075)

*CV*_*A*_ and *h*^*2*^ estimates when permanent environment effects were included in the models (as in Table 1) and when the permanent environment effect was removed. Refer to Table 1 for more details about sample sizes.

All variance estimates were conditioned by sampling date, age, and sex with the inclusion of fixed effects for these terms. Sampling date significantly affected blood mercury across all treatment levels (*p* < 0.01). Age at time of sampling affected blood mercury accumulation in the 0.3 (*p* = 0.002), 0.6 (*p* = 0.030), and 1.2 (*p* = 0.034) ppm dietary mercury treatments. Females had lower levels of mercury accumulation in their blood than males in the 0.3 (*p* < 0.001) and the 1.2 (*p* < 0.001) ppm dietary mercury treatments.

## Discussion

Blood mercury accumulation varied substantially among individuals at all dietary mercury treatments, yet was repeatable for individuals across repeated measurements. Genetic influence (i.e. “heritability”) on mercury accumulation was non-linear with increasing dietary mercury exposure, and we observed significant gene-by-environment interactions. A high genetic influence on blood mercury accumulation was observed at the intermediate dietary mercury concentrations of 0.6 and 1.2 ppm mercury (*h*^*2*^ = 0.458 and 0.341, respectively, Table 1). The highest mercury treatment, 2.4 ppm, also showed substantial heritability (*h*^*2*^ = 0.354, Table 2) when we removed the potentially confounding common permanent environment effect from our quantitative genetic models; however the heritability estimate for the lowest dietary-mercury treatment, 0.3 ppm, still remained low. These analyses support the conclusion that there is a non-linear genetic influence on blood mercury, with genetic variance being least when mercury exposure was also lowest. Perhaps more convincingly, our data support a robust conclusion that there is substantial genetic variance to blood mercury levels in our captive zebra finches. Mean-scaled measures of additive genetic variation (*CV*_*A*_) for the 0.6 and 1.2 ppm mercury dietary treatments exceeded *CV*_*A*_ values reported for most physiological, ornamental, and morphological traits in a recent review of quantitative genetics in the zebra finch [53]. Overall, these results indicate that there is substantial genetic variation for factors that lead to sub-lethal accumulation of mercury in the blood of these birds when the birds are exposed to mercury levels that could occur at a contaminated site.

Additional fixed effects are often fitted in Animal Models in order to separate influences of the environment from additive genetic effects [69], and thus provide better estimates of variance components. Fixed effects for sampling date, sex, and age were included in our models of blood mercury accumulation. The inclusion of fixed effects for sample date significantly improved all models of blood mercury accumulation, and age had a significant influence in all models except the 2.4 ppm mercury treatment. Estimates of additive genetic variance increased in all models after conditioning for the effect of sampling date; the further inclusion of age did not raise estimates of additive genetic variation. This suggests that both date and age reflect temporal differences in mercury accumulation over the course of this study; however, the factors responsible for differences in mercury accumulation with respect to date and age could not be determined. Variation among batches of mercury-dosed food is unlikely to explain differences with respect to sampling date, as measures of quality assurance for food preparation indicate high consistency of mercury concentrations (99–102% of desired concentrations) between batches. Subtle changes in the environmental conditions experienced by offspring, which, upon independence were moved from their parent’s cage to an aviary where they lived in large flocks, may have contributed to the variation due to both sampling date and age. In this case, the inclusion of date as a fixed effect may have conditioned for variation in environmental changes associated with movement to an aviary that allowed for greater social interaction and flight activity compared with the smaller parental breeding cages. It is also possible that the observed effects of age could relate to changes in mercury accumulation with life stage, though all birds were sampled as adults. Sex had significant effects on blood mercury accumulation for all treatments except at the 2.4 ppm dose. Females had lower mercury accumulation than males. Mercury excretion into eggs may explain lower accumulation in females; the effect was larger when offspring (which are too young to lay eggs) were excluded from the model. This result is consistent with other studies, which have reported lower mercury concentrations in females as a result of egg-laying [72,73]. The lower heritability estimate in the 2.4 ppm mercury treatment compared with the 0.6 ppm treatment may be the result of a reduced number of offspring produced by females in this treatment (Table 1). Using data from previous studies [3,22,59], we also explored whether individual body mass could explain variation in blood-mercury concentrations. Body mass could reflect individual differences in foraging activities and abilities to process and digest food. However, we could not find any statistical associations between body mass and blood-mercury concentrations, even though we had robust sample sizes in many of these comparisons (0.3ppm treatment, Pearson *r*_22_ = 0.270, *p* = 0.213; 0.5ppm treatment, *r*_67_ = -0.128, *p* = 0.297; 0.6ppm treatment, *r*_27_ = 0.227, *p* = 0.246; 1.0ppm treatment *r*_18_ = -0.420, *p* = 0.073; 1.2ppm treatment, *r*_22_ = 0.124, *p* = 0.572; 2.4ppm treatment, *r*_8_ = -0.297, *p* = 0.437).

A lack of general lack of genetic influence on mercury accumulation in both the lowest dietary mercury treatments may be the result of physiological thresholds which limit tolerance to mercury. Below the threshold where mercury toxicity negatively affects individual health, the energetic cost of a response to mercury may outweigh the benefits of tolerance. This hypothesis is consistent with research on mercury detoxification in wild birds; Eagles-Smith et al. [47] reported a threshold for mercury demethylation in waterbird livers where demethylation occurred only when liver mercury concentrations increased above 8.51 ± 0.93 ppm. Thresholds of demethylation have not yet been demonstrated in the zebra finch (or any songbird), and it is unclear if this effect is responsible for low genetic contribution to blood mercury accumulation in the 0.3 ppm treatment group. Future captive-dosing studies could investigate the potential for demethylation thresholds in zebra finches and the potential co-variation between liver detoxification of mercury and blood mercury accumulation.

In addition to partitioning phenotypic variation into sources of genetic and environmental variation, the prediction of the evolutionary potential of quantitative traits is a main goal of quantitative genetics [66]. In order for a trait to evolve under selection it must be both variable and heritable. Variation in mercury accumulation was highly heritable for the 0.6 and 1.2 ppm mercury treatments, and likely quite high at 2.4 ppm also. The repeatability of blood mercury accumulation in individuals may make this more stable as a trait for selection. Taken together, there is evidence of substantial genetic variation in blood mercury accumulation within dietary mercury treatments that could be acted on by selection. A concurrent analysis of the same captive population fed the same mercury concentrations demonstrated that mercury represents a selective pressure by reducing reproductive success in a dose-dependent manner from 16% and 50% relative to finches on control diets [3]. For the next generation (i.e., those that survived to breed under the challenge of mercury exposure) reproductive success was higher than in the previous generation [3], likely as a result of an adaptive response to selection for mercury tolerance.

If similar genetic variation for mercury accumulation exists in wild populations, persistent selection could result in genetic differentiation between stable populations at contaminated and reference locations. The dietary mercury treatments used in this study at 0.3, 0.6, and 1.2 ppm mercury span the range of dietary levels of exposure that songbirds experience at contaminated sites [7]. Studies in free-living songbirds have also revealed evidence of reduced reproduction in individuals living on mercury-contaminated sites [17]. Adaptation to mercury contamination in wild populations, either in the form of increased mercury mitigation (limited uptake, excretion pathways) or decreased sensitivity, could have consequences for biomagnification up food webs and conservation of populations. If there is increased mercury mitigation, individuals could reduce systemic mercury levels through increased deposition into feathers or eggs. In addition to these mechanisms, or alternatively, detoxification pathways could sequester biologically inert mercury in the liver; mercury stored in the liver in the form of mercuric selenide would be less readily bioaccumulated by predators [46]. If there is a reduction in mercury bioaccumulation, less mercury would be available to biomagnify up the food chain. Hence, increased mercury mitigation by individuals would likely decrease the amount and concentration of mercury in higher trophic levels of an ecosystem.

However, the evolution of mercury tolerance could result in a decreased sensitivity to the numerous detrimental effects of mercury. In the population where decreased sensitivity has evolved there may be lower risks of population decline or extinction, as individuals will be more tolerant to mercury. However, the evolution of a decreased sensitivity to the toxic effects of mercury could result in individuals tolerating and accumulating more mercury in their tissues over their lifetime, thus increasing the risk to the next trophic level in an ecosystem, as the predators will consume individuals from this more-tolerant population. Hence, under this scenario, we predict the potential for increased biomagnification of mercury at higher trophic levels, which could lead to greater mercury toxicity in predators (unless they adapt too), including the possibility of higher risks for hunters who consume wild-caught waterfowl, which can accumulate high levels of mercury at contaminated locations [74]. Migration has been linked to the transport of mercury and mercury-tolerant individuals could intensify the movement of mercury out of contaminated areas. Seabird-mediated mercury transport into high arctic ponds accounted for a 25-fold increase in mercury concentration compared to locations unused by seabirds [75]. Waterfowl exposed to mercury on the South River in Virginia, USA, have been collected by hunters as far as 1,054 km away [74].

The evolution of mercury-tolerance and mitigation factors may itself pose a risk to populations if the mechanisms of mitigation and tolerance are costly. For example, increased mercury deposition into eggs may increase embryonic mortality [10]. Similarly, mercury tolerance may impose a cost if the mechanisms associated with tolerance are energetically expensive, which could be likely as there may be up-regulation of cellular transport and demethylation pathways with increased tolerance.

It is also possible that wild populations would not adapt in response to mercury. Wild birds may not show a similar pattern of genetic influence on mercury accumulation as observed in this captive population. Estimates of heritability can vary over time and under different environmental conditions [76]. Likewise, selection pressures can vary, making long-term prediction of microevolutionary change uncertain. Gene flow among contaminated and uncontaminated populations could also slow or prevent the evolution of mercury tolerance. Lack of an adaptive response could imply persistent detrimental effects of mercury toxicity in populations that do not acquire tolerance. Alternatively, the cost of mercury tolerance could be too high for tolerance to evolve, or variation in exposure as populations move between contaminated and non-contaminated areas could induce a selective pressure that is insufficient to cause adaptive change.

As global mercury pollution increases and bird populations decline there is potential for wild populations to evolve in response to mercury toxicity, hence we feel it is prudent to attempt to quantify the evolutionary potential of populations, especially as the evolution of mercury tolerance has implications for risk assessment and population conservation. It would be possible to take the Animal Model approach we describe here and apply it to known pedigrees of free-living organisms to better understand the evolvability of factors related to mercury tolerance. In addition to quantifying heritability in this manner, researchers should study associations of heritable traits with fitness variation in their populations. Any non-zero associations can indicate a current selection pressure and predict generational changes in traits. It would then be possible to track the predicted trait changes over subsequent generations to examine whether the population is adapting to mercury exposure.
